# The Silver Lining in a Time of Crisis: The Foundation of the Society for the Study of Neuroprotection and Neuroplasticity (SSNN) Conference Proceedings Go Virtual

**DOI:** 10.25122/jml-2020-1006

**Published:** 2020

**Authors:** Dafin Muresanu, Stefan Strilciuc

**Affiliations:** SSNN

As we approach the one-year mark since our unwanted acquaintance with the novel coronavirus, the scientific community is forced to adapt to widespread restrictions across the life sciences research landscape, spanning from institutional shutdowns to city-wide curfews and travel bans. As a result, many conference proceedings globally have been postponed, delayed or cancelled.

Difficult times ask for creative and courageous solutions to complex issues. In the interest of the health, safety and well-being of all registered attendees and the general public, the Society for the Study of Neuroprotection and Neuroplasticity (SSNN) Board of Directors has decided to migrate all upcoming events that require in-person attendance to a virtual environment. We are committed to supporting public health authorities globally in their effort to slow and contain the spread of COVID-19. We must stay positive, healthy and united more than ever to overcome this crisis.

Our long-established events - the 10^th^ European Teaching Course on Neurorehabilitation (4-5 September 2020), the 15^th^ International Summer School of Neurology (6-7 September 2020) and the 4^th^ EAN Task Force on Rare Neurological Diseases Teaching Course (8 September 2020) - will be offering excellent opportunities for education, dissemination of scientific research, as well as the exchange of best practices, through a brand new online platform that has been tailored to promote lively interaction between participants (Figure 1).

**Figure 1:**
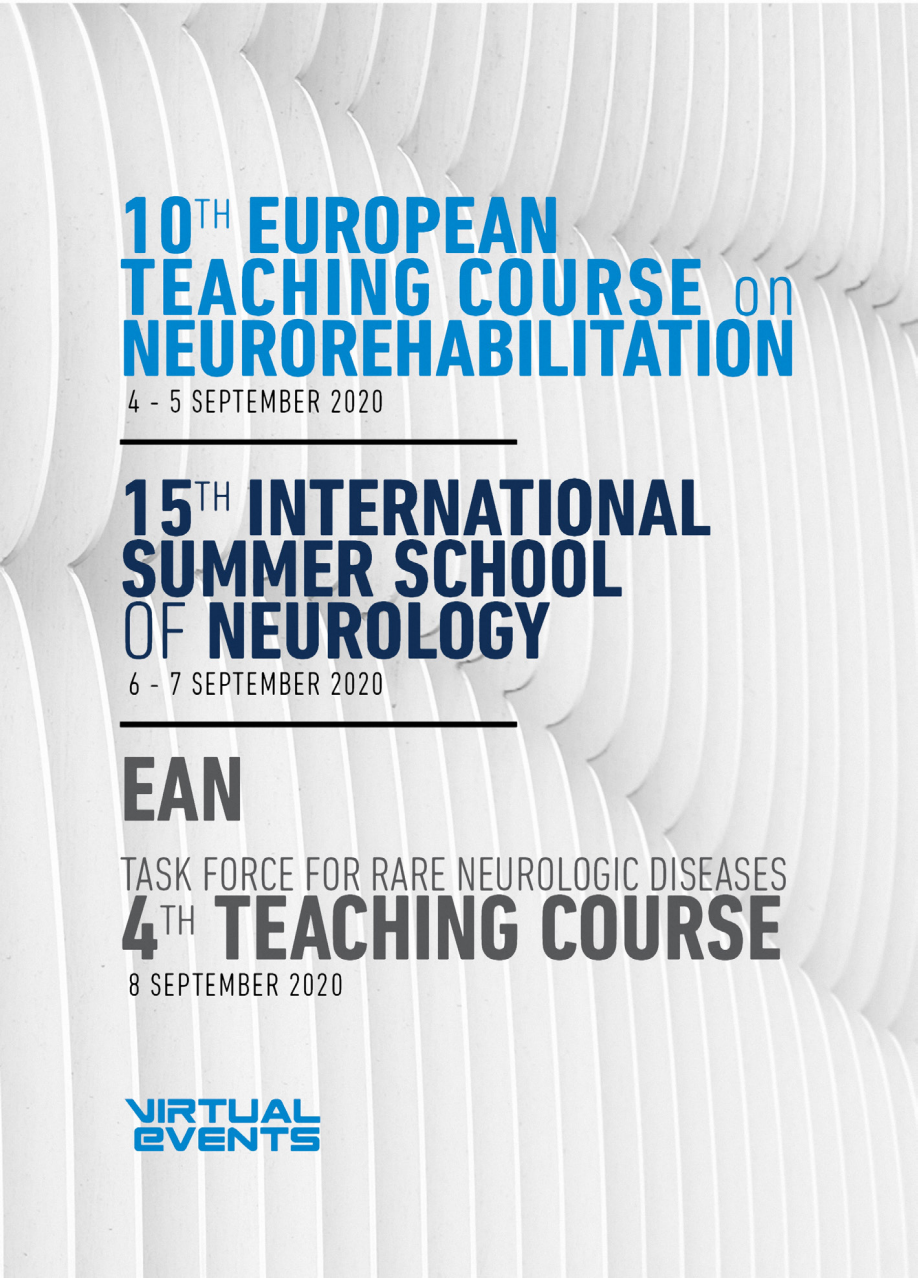
SSNN Virtual Events organized in September 2020.

## 10^th^ European Teaching Course on Neurorehabilitation

The Teaching Course is among the most prestigious and long-lasting events organized by the SSNN. After nine successful events, the first virtual teaching course promises to rise to participants’ expectations to serve as a platform to disseminate and exchange up-to-date scientific information in the field of neurorehabilitation, as well as to provide a space for teaching-oriented workshops. Each year, the event reaches an audience with an interest in this steadily expanding and exciting field (i.e., physicians, nurses, therapists, public health professionals, and more).

The overarching topic of this event is assessing where neurorehabilitation stands today in Europe and beyond, as well as identifying future perspectives for education, science and service delivery in the field. The Scientific Program Committee, led by prof. Volker Homberg, Vice-President of the European Federation of Neurorehabilitation Societies (EFNR) and Secretary-General of the World Federation of Neurorehabilitation Societies (WFNR), has carefully curated a program based on submissions from our top-notch international faculty.

The event’s objectives are (1) to advance the development and improve the quality of neurorehabilitation in Europe, (2) to stimulate the collaboration between neurorehabilitation professionals, (3) to facilitate the exchange of knowledge and scientific research between clinicians with interest in neurological rehabilitation, and (4) to contribute to the development of cooperation and communication networks between national and international neurorehabilitation societies.

## 15^th^ International Summer School of Neurology

The International Summer School’s mission is to provide a platform where young neurologists-in-training can interact with an internationally renowned faculty with vast expertise in both basic and clinical neurosciences. The idea for this educational event dates back to 2005 when alongside professors Natan Bornstein (Israel), and Ovidiu Bajenaru (Romania), we sought to address the need of young specialists’ and practitioners’ to be connected with the latest developments in the complex field of neurosciences. Ever since, we have been developing a dynamic environment to facilitate this type of synergy. In this year’s virtual event, international experts will cover topics like blood-brain barrier research, secondary stroke prevention, the latest advances in neuroimaging and stroke, epilepsy, neurodegenerative disorders, and movement disorders.

## 4^th^ EAN Task Force on Rare Neurological Diseases Teaching Course

The EAN Teaching course on rare neurological disorders (RNDs) is an exceptional event we have been organizing with the support of professor Antonio Federico (Italy). From a neuro-epidemiological perspective, rare diseases have significant public health impact due to their collective large number and diversity. The task of providing care for over 5,000 documented diseases that are considered to be rare worldwide is a daunting experience. While significant progress has been made in recent years with understanding and mapping rare diseases, providing early diagnosis and valid treatment options for patients with such afflictions is still a significant challenge. RNDs are vastly underdiagnosed, and effective treatment is often lacking. The European Academy of Neurology (EAN) Scientific Committee has established a task force intending to help patients with RNDs and their families through strategies to facilitate earlier diagnosis, timely management and coordinating research.

The current virtual events benefit from the active support of some of the longstanding partners like the European Federation of Neurorehabilitation Societies (EFNR), World Federation of Neurorehabilitation Societies (WFNR), the European Academy of Neurology (EAN), “Iuliu Hatieganu” University of Medicine and Pharmacy from Cluj-Napoca, Romania, the RoNeuro Institute for Neurological Research and Diagnostic, and the Journal of Medicine and Life.

## This year’s silver lining

The 2020 SSNN virtual events will set the stage for five days of intensive talks and debates between over a thousand participants from 25 countries on a broad range of problems in neurosciences. SSNN will also be showcasing an exciting avenue for development – our collaboration with NeurotechEU (www.theneurotech.eu) – the European University of Brain and Technology. NeurotechEU is a newly funded European University that hopes to build a trans-European network of excellence in brain research and technologies to increase the competitiveness of European education, research, economy and society. The university alliance is comprised of Radboud Universiteit (The Netherlands, project coordinator), Universidad Miguel Hernández (Spain), Karolinska Institutet (Sweden), Rheinische Friedrich-Wilhelms-Universität Bonn (Germany), Boğaziçi Üniversitesi (Turkey), Oxford University (Great Britain), the Iuliu Hatieganu University of Medicine and Pharmacy Cluj-Napoca (Romania), and Debreceni Egyetem (Hungary).

Despite our collective unrest caused by this unprecedented global health emergency, we must stand our ground and power through this turmoil - together. Our previous lives may not be up for resuming, but it is definitely in our hands to pave the path for a brighter future.

